# The predictive effect of body mass index on type 2 diabetes in the Norwegian women and cancer study

**DOI:** 10.1186/1476-511X-13-164

**Published:** 2014-10-24

**Authors:** Mashhood Ahmed Sheikh, Eiliv Lund, Tonje Braaten

**Affiliations:** Institute of Community Medicine, University of Tromsø, N-9037 Tromsø, Norway

**Keywords:** BMI, Type 2 diabetes, Norway

## Abstract

**Background:**

Several studies have analyzed the association of body mass index (BMI) with either the prevalence or incidence of type 2 diabetes (T2D), but no study from Europe or North America has yet analyzed and compared the association of BMI with both incident and prevalent T2D cases.

**Methods:**

Stratified logistic regression was used to calculate odds ratios (OR), and stratified Cox proportional hazards regression was used to calculate hazard ratios (HR) of the effect of BMI on the prevalence, and incidence of T2D. Wald chi-square statistics were applied when comparing the risk estimates.

**Results:**

Among prevalent T2D cases, overweight women (BMI 25–29.9 kg/m^2^) had an OR of 2.83 (95% confidence interval [CI], 1.92-4.18) and obese women (BMI ≥30 kg/m^2^) had an OR of 12.12 (95% CI, 8.32-17.68) when compared with normal weight women (BMI <25 kg/m^2^). Among incident T2D cases, overweight women had a HR of 5.01 (95% CI, 3.59-6.98) and obese women had a HR of 15.99 (95% CI, 11.39-22.46) when compared with normal weight women. After stratification by level of physical activity, and adjustment for age, smoking status, and education level, the Wald chi-square statistic for BMI was 180.90 for prevalent T2D cases, and 262.03 for incident T2D cases.

**Conclusion:**

The predictive effect of BMI was found to be stronger for T2D incidence than T2D prevalence.

## Background

Type 2 diabetes (T2D) is a chronic disorder of carbohydrate, fat, and protein metabolism. Approximately 60 million people in Europe have diabetes [1], and 90% of the diabetes patients worldwide have T2D [2]. T2D is largely the result of excess body weight and physical inactivity [2]. There is ample evidence that obesity is a major risk factor for T2D, as obesity is associated with the rise of insulin resistance in the body, resulting in the development of T2D [3–6]. The prevalence of diabetes has been increasing in Norway [7, 8]. The Nord-Trøndelag Diabetes Study showed a diabetes prevalence of 0.6% and 2.0% for women aged 40–49 and 50–59 years, respectively, in 1984–1986 [7], and the Nord-Trøndelag Health Survey (HUNT) showed a prevalence of 0.9% and 2.1%, respectively, in 1995–1997 [9]. The joint relationship of body mass index (BMI) and physical activity with diabetes remains unclear [10–12]. Some research indicates that physical activity is associated with T2D independent of obesity [13], but most studies indicate that the relationship between physical activity and T2D weakens when BMI is taken into consideration [12, 14–16].

The Tehran Lipid and Glucose Study (TLGS) included fasting plasma glucose screening and 2-hour post challenge plasma glucose screening to identify T2D cases in 1999–2001, 2002–2005, and 2005–2008, and found higher odds ratios (OR) for BMI among incident than prevalent T2D cases [17]. However no study from Europe or North America was found where the association of BMI with both incident and prevalent T2D cases was analyzed. Therefore, using data from the Norwegian Women and Cancer (NOWAC) Study, we performed a cross-sectional analysis of data collected in 1998, and a prospective cohort analysis of data collected between 1998 and 2005, and compared the OR estimates of BMI in relation to T2D prevalence and incidence.

## Methods

### Study population

The NOWAC Study is a prospective nationwide study which started in 1991 [18], and contains data from 170,000 women. Participants were randomly selected from the National Population Register of Norway. The external validity of the study has been published elsewhere [19]. NOWAC Study participants are assumed to be representative of the female Norwegian population in the corresponding age groups. The detailed sample characteristics of the NOWAC Study are described elsewhere [19], and updated information on the NOWAC Study is accessible on the website [18].

Out of the 170,000 women enrolled in the NOWAC Study, 33,919 completed the questionnaires sent in 1998 and 2005 (age: 47.7 years ±4.3, BMI: 24.4 kg/m2 ± 3.8, education level: 12.5 years ±3.2). After exclusion of 2617 participants with missing values, the study sample consisted of 31,302.

### Questionnaire and classification

As T2D typically affects people over 40 years of age [20], in the present analysis prevalent T2D cases were defined as participants who reported a diabetes diagnosis in the 1998 questionnaire, and were 40 years of age or over at the time of diagnosis. If the participants gave birth to a child the same year, or the year preceding diabetes diagnosis, it was assumed that they had gestational diabetes. Only one woman fulfilled the criteria for T2D and gestational diabetes, and was considered to have gestational diabetes only.

Incident T2D cases were defined as participants who reported a T2D diagnosis between 1998 and 2005, and were 40 years of age or over at the time of diagnosis (Table 1). For women without a diabetes diagnosis, person-years were calculated from the time of the 1998 questionnaire until 2005, when the last questionnaire was completed. For incident T2D cases, person-years were calculated from the time of the 1998 questionnaire until year of diabetes diagnosis.

**Table 1:** **General characteristics of the study sample (n = 33,919)**

	Baseline cohort N = 33,919		Incident T2D cases		Prevalent T2D cases	
	N (%)	Mean (SD)	N (%)	Mean (SD)	N (%)	Mean (SD)
**Age (years)**		47.7 (4.3)		48.9 (4.3)		50.3 (3.9)
40-44	9926 (29.3)		70 (21.4)		25 (12.3)	
45-49	11382 (33.6)		98 (30.0)		43 (21.1)	
50-54	10849 (32.0)		137 (41.9)		107 (52.5)	
55-59	1762 (5.2)		22 (6.7)		29 (14.2)	
BMI*‡§		24.4 (3.8)		29.7 (5.4)		29.8 (6.3)
Normal weight (<25 kg/m^2^)	21553 (64.6)		55 (17.6)		47 (23.4)	
Overweight (25–29.9 kg/m^2^)	9106 (27.3)		126 (40.3)		64 (31.8)	
Obese (≥30 kg/m^2^)	2709 (8.1)		132 (42.2)		90 (44.8)	
Education level (duration in years)*‡§		12.5 (3.2)		11.7 (3.1)		11.4 (2.9)
Primary/Intermediate (0–9)	6736 (20.1)		91 (27.9)		63 (31.2)	
Secondary (10-12)	12102 (36.1)		125 (38.3)		83 41.1)	
University (13-16)	10226 (30.5)		88 (27.0)		36 (17.8)	
Postgraduate and above (17+)	4460 (13.3)		22 (6.7)		20 (9.9)	
Physical activity level*‡§		5.6 (1.7)		4.7 (1.8)		4.9 (1.9)
Low	3686 (11.5)		76 (25.5)		41 (21.4)	
Medium	24229 (75.5)		200 (67.1)		133 (69.3)	
High	4186 (13.0)		22 (7.4)		18 (9.4)	
**Smoking status**						
Never smoker	13763 (40.6)		124 (37.9)		71 (34.8)	
Former smoker	10582 (31.2)		106 (32.4)		70 (34.3)	
Current smoker	9574 (28.2)		97 (29.7)		63 (30.9)	
**Age at diagnosis (years)**				53.1 (4.5)		46.3 (4.2)
40-44			10 (3.1)		75 (36.8)	
45-49			69 (21.1)		70 (34.3)	
50-54			107 (32.7)		59 (28.9)	
55-59			117 (35.8)		0 (0.0)	
60-64			24 (7.3)		0 (0.0)	

*Cohort size was 33,919, but because of missing values, the numbers for some variables do not add up to 33,919.

‡The total number of incident cases of T2D was 327, but because of missing values, the numbers for some variables do not add up to 327.

§The total number of prevalent cases of T2D was 204, but because of missing values, the numbers for some variables do not add up to 204.

Self-reported information on height and weight was used to calculate BMI (in kg/m^2^). BMI was categorized into three groups: normal weight (BMI <25 kg/m^2^), overweight (BMI 25–29.9 kg/m^2^) and obese (BMI ≥30 kg/m^2^). Both continuous and categorical BMI variables were used in the analyses.

Smoking status was derived from the replies to two questions in the 1998 questionnaire: ‘Have you ever smoked?’ (yes, no), and ‘Do you smoke on a daily basis at the moment?’ (yes, no). Women who answered ‘no’ to the former were categorized as ‘never smokers’. Those who answered ‘yes’ to the former, and ‘no’ to the latter, were categorized as ‘former smokers’, and those who answered ‘yes’ to both questions were categorized as ‘current smokers’.

A 10-category scale measured the level of self-reported physical activity in the 1998 questionnaire, the validity of which has been reported [21]. Responses to questions about physical activity were used to assign a category of physical activity: low [1–3], medium [4–7], and high [8–10]. Participants also reported education level (duration in years), and age (years).

### Statistical analysis

All analyses were conducted using SPSS version 18. Means (standard deviation, SD) were calculated for all continuous variables, and the percentage of participants in each category was determined for all categorical variables. General characteristics of the data are presented as means with SDs and frequencies, respectively (Table 1).

To estimate the predictive effect of BMI on the incidence and prevalence of T2D, stratified logistic and stratified Cox proportional hazards regression were used. To assess the linear trend, the continuous variables (BMI, education level, and physical activity) were used. To assess the predictive effect of BMI, the normal weight level was used as a reference in stratified logistic regression and stratified Cox proportional hazards regression analyses. A *p* value of <0.05 was considered statistically significant. More than 5% change in beta coefficients was used as the cutoff to identify possible confounders, and by this method education level, physical activity, and smoking status were identified as confounders of the association between BMI and T2D. All independent variables were tested for pairwise interaction with BMI with logistic and Cox proportional hazards regression models. A *p* value of <0.05 was considered significant for identifying possible interactions. The relationship between BMI and T2D was not found to be linear in our analysis (results not shown), and so the categorical variable of BMI was used in the final models instead. In the final model, the estimates of the effects of BMI are presented with 95% confidence intervals (CI). ORs and hazard ratios (HR) are reported. An OR can be interpreted as a relative risk (RR) when the disease prevalence is low [22]. Wald chi-square statistics were reported to present the overall predictive effect of BMI on the development of T2D, stratified by physical activity for comparison between T2D prevalence and incidence. Both adjusted and unadjusted estimates are presented.

### Ethical approval

The NOWAC Study was approved by the Regional Committee for Medical and Health Research Ethics (REK). All women gave written informed consent.

## Results

**Figure 1:**
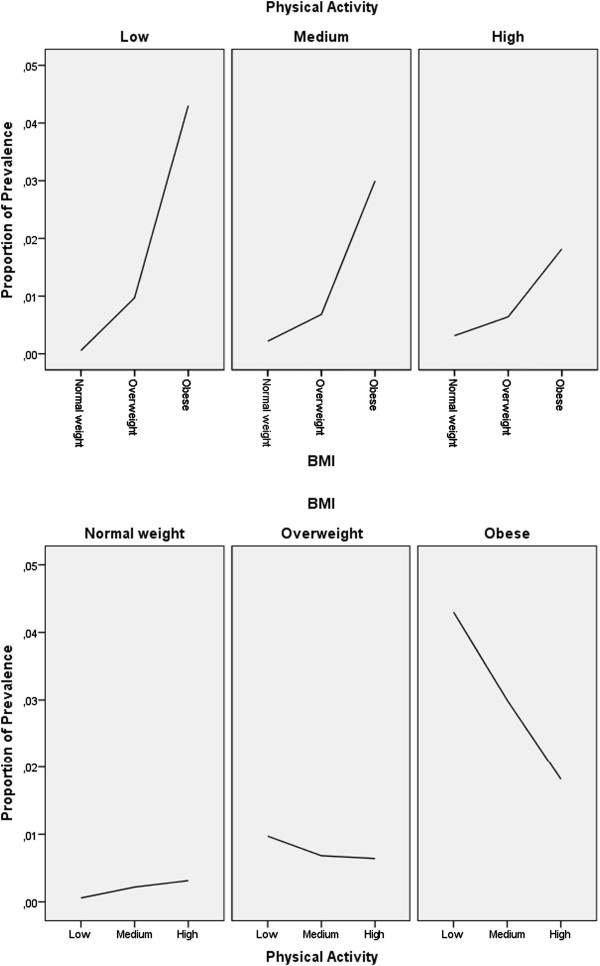
**Proportion of T2D incidence by BMI and level of physical activity.**

**Figure 2:**
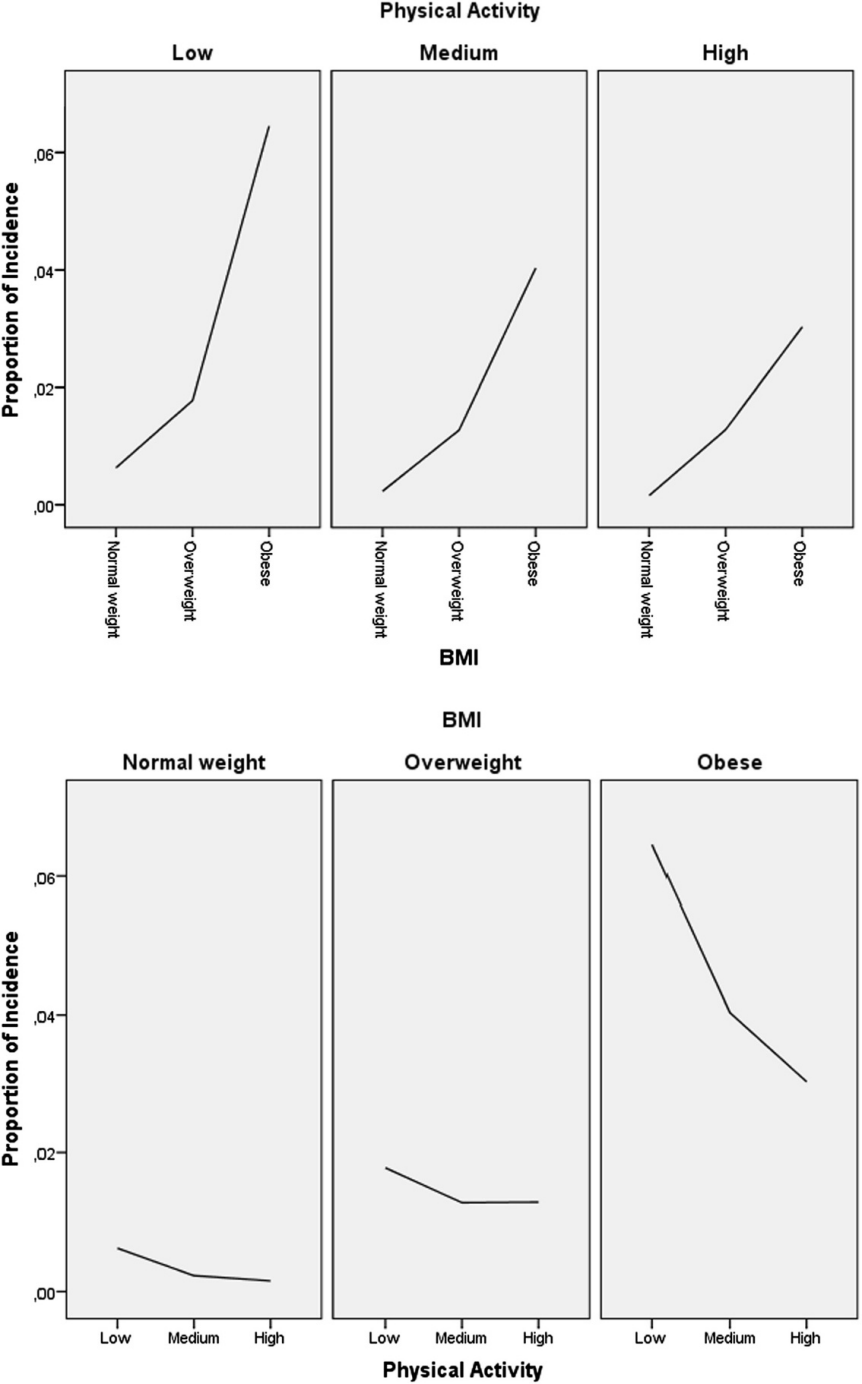
**Proportion of T2D prevalence by BMI and level of physical activity.**

T2D prevalence was assessed in 33,919 women, of whom 204 were classified as prevalent T2D cases. T2D incidence was assessed in 33,714 women over 7 years of follow-up, and 327 were classified as incident T2D cases. The characteristics of the study sample, i.e., the baseline cohort, and prevalent and incident T2D cases, are shown in Table 1. Compared with the baseline cohort, prevalent and incident T2D cases had higher BMI, lower education level, and lower level of physical activity. The mean BMI of the baseline cohort was 24.4 kg/m^2^. A higher proportion of incident T2D cases were overweight and obese (combined), compared to prevalent T2D cases. The mean BMI of prevalent T2D cases was slightly higher than that of incident T2D cases (29.8 vs 29.7 kg/m^2^). The majority of women in the baseline cohort had a normal weight level, while the majority of incident and prevalent T2D cases were obese. Compared with prevalent T2D cases, incident T2D cases were on average younger, had a slightly lower BMI, a slightly higher education level, and a slightly lower level of physical activity (Table 1).An interaction between BMI and physical activity was observed in T2D. Figures 1 and 2 show that the effect of BMI on the incidence and prevalence of T2D changes according to the level of physical activity, and the models were therefore stratified by physical activity (Figures 1 and 2). Physical activity was identified as the effect modifier, and the three categories of physical activity were employed as strata variables in the stratified logistic and stratified Cox proportional hazards regression analysis.

The independent effect of BMI on T2D in both unadjusted and multivariate-adjusted models is presented in Table 2. Each group had a statistically significantly increased risk of prevalent and incident T2D when compared with normal weight women: overweight women had an OR of 2.83 (95% CI, 1.92-4.18), and obese women had an OR of 12.12 (95% CI, 8.32-17.68) for prevalent T2D. Compared with normal weight women, overweight women had a HR of 5.01 (95% CI, 3.59-6.98), and obese women had a HR of 15.99 (95% CI, 11.39-22.46) for developing incident T2D. After adjustment for age, smoking status, and education level, the Wald chi-square statistics for prevalence and incidence were 180.90 and 262.03, respectively, showing that BMI has a stronger predictive effect on T2D incidence than prevalence.

**Table 2:** **Estimates of BMI stratified by level of physical activity for prevalent and incident T2D cases**

	OR (95% CI) and Wald ***χ*** ^2^		HR (95% CI) and Wald ***χ*** ^2^	
	Prevalence (unadjusted)	Prevalence (adjusted)*	Incidence (unadjusted)	Incidence (adjusted)*
Normal weight	1.00 (ref.)	1.00 (ref.)	1.00 (ref.)	1.00 (ref.)
Overweight	3.24 (2.20-4.76)	2.83 (1.92-4.18)	5.09 (3.66-7.07)	5.01 (3.59-6.98)
Obese	14.20 (9.80-20.59)	12.12 (8.32-17.68)	16.46 (11.79-22.97)	15.99 (11.39-22.46)
Wald *χ* ^2^	207.50	180.90	276.64	262.03
*p* for trend	p < 0.001	P < 0.001	p < 0.001	p < 0.001

*Adjusted for age, education level and smoking status.

## Discussion

In this study, we estimated the cross-sectional and prospective relationship between BMI and T2D in a nationally-representative sample of middle-aged women in Norway. We observed that BMI is a stronger predictor of incident T2D (reported between 1998 and 2005), than prevalent T2D (reported in the 1998 questionnaire). Overall, BMI had a stronger predictive effect on T2D incidence than T2D prevalence.

The risk of T2D prevalence was weaker than that for incidence, probably because the prevalent cases may have reduced their weight by exercise, physical activity, or diet after diagnosis.

Previous validation studies of self-reported height and weight show that participants tend to overestimate their height [23], while they tend to underestimate their weight [23–25]. This can affect the strength of the association between BMI and T2D, but not the trend. The large sample size and a relatively long follow-up time are important strengths of our study.

Several studies have adjusted for age [14, 26–34], smoking status [14, 27–31, 34], and education level [31, 35] in their models while using BMI to predict the risk of T2D. We identified the same confounders, and included them in our final models. The World Health Organization estimates that a BMI of >25 kg/m^2^ may account for 65%-80% of new diabetes cases [36], which is in accordance with our study. Previous research has shown that physical inactivity plays a major role in the etiology of both T2D and obesity. BMI was negatively correlated with physical activity in our study population (data not shown), in accordance with previous studies [34, 37, 38]. Possible explanations for the role of physical activity as an effect modifier in this research may be that physical activity increases sensitivity to insulin [39], and can result in weight loss [40].

Previous evidence from large cohort studies suggests that the relationship between BMI and diabetes may not be linear, and the same was observed in our study. In comparison with previous studies, despite differences in the groups of confounders in the model [14, 26, 31–34], study designs [17, 31, 33], and methodology [32, 34], a similar pattern of association between BMI and T2D was observed. The results from the NHANES III [26] study showed that among women aged less than 55 years, the risk of T2D was relatively less for women with BMI 30.0-34.9 kg/m^2^ than for women with BMI 25.0-29 kg/m^2^. This is in contrast to our study, although we only used one category [BMI ≥30 kg/m^2^). However, for women aged 55 years or older, the NHANES III study showed an increased risk of T2D with increasing BMI. In general the association between BMI and T2D prevalence in our study is much higher than in other studies [26, 31].

The HUNT Study [27] from Norway reported estimates of the effect of BMI on T2D incidence during 11 years of follow-up. T2D cases were established by clinical history and presence or absence of the anti-GAD antibody. T2D incidence was assessed from 1984–1986 to 1995–1997, as compared to 1998–2005 in our study. Still, the estimates were very similar, showing that despite the use of clinical history and presence or absence of the anti-GAD antibody to determine T2D incidence, our study yielded similar risk estimates. Nord-Trøndelag County, where the HUNT Study was carried out, is considered representative of the general population of Norway [7], whereas our study population represents middle-aged women in Norway. Nevertheless, there are some similarities between the results of the HUNT study and our study. The mean BMI of prevalent T2D cases in our study was similar to the HUNT study during 1995–1997 [9]. The cohort size was similar, as was the proportion of participants with BMI ≥30 kg/m^2^ at baseline. Also the proportion of diabetic participants with a BMI of ≥30 kg/m^2^ in 1995–1997 was similar to that in our study.

Another study [31] from the US, using self-reported information on diabetes diagnosis, weight, and height with telephonic interviews analyzed the OR of BMI for prevalent diabetes. No distinction was made between different types of diabetes, or between men and women. Compared with normal BMI, the OR for BMI 25–29.9 kg/m^2^, BMI 30–39.9 kg/m^2^, and BMI .40 kg/m^2^ were 1.59 (95% CI: 1.46-1.73), 3.44 (95% CI: 3.17-3.74), 7.37 (95% CI: 6.39-8.50) respectively. The model was adjusted for age, education, smoking, sex, and race or ethnicity. Regardless of the use of different cut off points in defining BMI levels, the OR’s are considerably lower as compared to our study.

A study from Finland [32] analyzed the BMI estimates for incident diabetes. The random sample of 35–64 year old men and women with no anti-diabetic drug treatment at baseline were followed for 10 years. The BMI was calculated using the height and weight measurements in a clinical examination. The diabetes diagnosis was established as the development of drug-treated diabetes using the information from the nationwide Social Insurance Institution drug register, and the FPG/FWBG/PG/WBG levels in the clinical examination. The interaction between the independent variables were not considered and sex was not included in the final model. The OR’s of BMI 25–30 kg/m^2^ for diabetes was not significant, while the OR’s for BMI >30 kg/m^2^ was 2.55 (95%CI: 1.10-5.92). The model was adjusted for age, waist circumference, use of blood pressure medication, history of high blood glucose, physical activity, and consumption of vegetables and fruits. In comparison, the results from our study show much stronger association of BMI levels with the prediction of type 2 diabetes in the incidence of diabetes.

Women’s Health Study (WHS) [14] from U.S assessed the predictive effect of BMI on the incidence of diabetes during 6.9 (mean) years of follow up. BMI was calculated from self-reported information on height and weight at the baseline. The type 2 diabetes diagnosis was established by annual self-reports by the respondents, and its validity was established. The mean BMI was 25.9 kg/m2 in 37878 women. Compared with BMI <25 kg/m2, the OR’s for BMI 25- < 30 kg/m^2^, and BMI ≥30 kg/m^2^ were 3.22 (95% CI: 2.69-3.87), and 9.06 (95% CI: 7.60-10.8) respectively. The model was adjusted for age, family history of diabetes, alcohol use, smoking status, hormone therapy use, hypertension, high cholesterol, dietary factors, randomized Women’s Health Study treatment groups, and physical activity. The relative risks of developing type 2 diabetes are in line with our research.

The Nurses’ Health Study [34] from U.S reported the estimates of BMI on the incidence of type 2 diabetes among female nurses aged 30–55 years. The follow up time was 16 years. The diagnosis of type 2 diabetes was established by sending follow up questionnaires biennially, and its validity was established in a subsample. Compared to BMI <23.0 kg/m^2^, the RR’s of BMI 23.0–24.9 kg/m^2^, BMI 25.0–29.9 kg/m^2^, BMI 30.0–34.9 kg/m^2^, and BMI ≥35.0 kg/m^2^ were 2.67 (95% CI: 2.13–3.34), 7.59 (95% CI: 6.27–9.19), 20.1 (95% CI: 16.6–24.4), and 38.8 (95% CI: 31.9–47.2) respectively. The model was adjusted for age, time, family history of diabetes, menopausal status, postmenopausal hormone therapy, dietary score, exercise, smoking, and alcohol consumption. The mean BMI was not reported, but the relative risks were substantially larger than our study.

The Framingham Offspring study from the US analyzed BMI estimates to predict the incidence of diabetes over 7 years of follow-up [33]. Despite having a higher mean BMI, and a similar follow-up time, the estimates were much weaker compared to our study. In comparison with most of the other studies [14, 32, 33], our study shows a much stronger association of BMI with the prediction of incident T2D. Only one study [17] was found where the estimates of BMI were reported for both the prevalence and incidence prediction. The TLGS showed higher ORs of BMI for incident diabetes mellitus than prevalent diabetes mellitus. Unlike our study, the data collected in the TLGS for the analysis of BMI and diabetes was not self-reported. Nonetheless, our study confirms that the same pattern of difference between prevalent and incident T2D can successfully be established using self-reported information. Our results further confirm the widely accepted hypothesis that BMI is a strong predictor of incident T2D, and that the relationship with physical activity cannot be ignored.

In conclusion, our study shows that maintaining a normal weight level is beneficial in preventing T2D. Our findings show a stronger predictive effect of BMI on T2D incidence than T2D prevalence. Overall the findings suggest that the majority of T2D cases can be prevented with weight loss.

## Abbreviations

BMI: Body mass index
T2D: Type 2 diabetes
OR: Odds ratio
HR: Hazard ratio
CI: Confidence interval
RR: Relative risk.
